# Investigating Water Balance as a Nutritional Determinant in Breastfeeding: A Comparative Study of Water Consumption Patterns and Influencing Factors

**DOI:** 10.3390/nu16132157

**Published:** 2024-07-06

**Authors:** Olga Malisova, Kyriaki Apergi, Emmanouil Niaos, Fotini Xenaki, Maria Kapsokefalou

**Affiliations:** 1 Department of Food Science and Technology, University of Patras, 2 G. Seferi St., 30100 Agrinio, Greece; kyapergi@upatras.gr; 2 Laboratory of Food Chemistry and Analysis, Department of Food Science and Human Nutrition, Agricultural University of Athens, 75 Iera Odos St., 11855 Athens, Greece; manosniaos@gmail.com (E.N.); xenakifotini@gmail.com (F.X.); kapsok@aua.gr (M.K.)

**Keywords:** water intake, breastfeeding, water balance, hydration

## Abstract

Background: Ensuring adequate hydration is critical for breastfeeding women, yet their water consumption patterns and hydration status is poorly understood. This study investigates the water consumption patterns and estimated water balance among women, practicing exclusive, mixed, and no breastfeeding methods. Methods: 529 healthy women completed the Nursing Water Balanced Questionnaire (N-WBQ). Participants were distributed across breastfeeding groups as follows: exclusive (39.7%), mixed (31.9%), and no breastfeeding (28.4%). Results: Significant differences were noted in water consumption patterns among breastfeeding groups regarding intake from beverages (*p* < 0.001), juices (*p* = 0.019), coffee (*p* < 0.001), and milk (*p* = 0.015). Water intake from liquids, except for drinking water (*p* < 0.001), juices (*p* = 0.024) and coffee (*p* < 0.001) differed significantly among groups in women with adequate total water intake based on recommendation, with exclusive breastfeeding mothers prioritizing plain water over other beverages. Total water loss (*p* < 0.001) and estimated water balance (*p* < 0.001) significantly varied among breastfeeding groups, with exclusive breastfeeding mothers to exhibit the lowest water balance (−475.36 mL/day), indicating potential dehydration risk. Apart from plain water, water from foods, coffee and milk significantly contributed to positive water balance. Conclusions: Our findings highlight a risk of dehydration in this population, while water consumption patterns are influenced by breastfeeding method, likely affected by varying lactational demands and lifestyle factors. Further research to develop more accurate and individualized methods for assessing water balance in breastfeeding women is needed.

## 1. Introduction

Hydration is essential during breastfeeding, as needs for water are increased. It is estimated that breast milk contains approximately 87% water with the milk produced at the beginning of the feeding (foremilk) to have the highest water concentration [1]. During the first six months of exclusive breastfeeding, milk production increases to an average of 750 mL per day [2]. In this period, high water needs increase the risk of dehydration in breastfeeding mothers, which can lead to decreased milk production, fatigue, muscle cramps, headaches, dry mouth, and nausea [3].

In Europe, the recommended total water intake for breastfeeding women, as suggested by European Food Safety Authority (EFSA), was based on theoretical evidence that breastfeeding women produce approximately 700 mL of milk per day. To compensate for this loss due to milk production, it was suggested that water intake should be increased by an additional amount of 700 mL per day. Thus, the total recommended daily water intake for breastfeeding women is 2700 mL [4].

Previous research, examining water intake in breastfeeding, has found that many women do not meet the recommended daily water intake. Two studies in Indonesia [5,6] and one in Mexico [7] revealed that almost 54% of breastfeeding women consumed less than the recommended daily amount of water. In another study in Beijing the percentage reached the 27% [8].

However, research on hydration status in breastfeeding women in Europe is limited. The large majority of available studies have been conducted in non-European countries, with different climate and seasonal variation. In most studies total water intake is usually estimated by considering only plain water and beverages yet excluding water from foods. Moreover, dietary patterns of breastfeeding women vary significantly among populations. This can significantly alter water consumption patterns, especially regarding water intake through foods and beverages.

The self-administrated Nursing Water Balanced Questionnaire (WBQ-N) is a new tool for evaluating water intake, defined as the total intake of water from all sources, encompassing plain drinking water, beverages (e.g., juices, soft drinks, tea, coffee, milk), and the water content present in foods, and water balance in breastfeeding women. The WBQ-N was based on the Water Balanced Questionnaire (WBQ) [9], which has been modified to include questions that reflect the specific dietary patterns, fluid intake behaviors, and unique hydration needs of breastfeeding women [10,11]. The use of this tool allows for a more accurate assessment of hydration status, by including water from all sources and facilitating the identification of women at risk for dehydration.

This study aimed to explore the estimated water balance in a sample of breastfeeding women in Greece, analyzing the factors that influence water intake and balance, taking into account the water intake from plain water, beverages, and foods, with the use of WBQ-N.

## 2. Materials and Methods

### 2.1. Study Design and Population

In this manuscript, we analysed data from a cross-sectional the study. Participants were selected based on specific criteria: they had to be healthy women aged 18–45 years, currently breastfeeding or having breastfed within the past six months. Based on their breastfeeding practices, participants were classified into three distinct groups: 1. exclusive breastfeeding: mothers who provided only breast milk to their infants, without the addition of any other liquids or solids, except for oral rehydration solutions, vitamins and medications in line with standard guidelines [12,13], 2. mixed breastfeeding: mothers who supplemented breast milk with formula or other solid or liquid foods [12], and 3. no breastfeeding: mothers who did not provide any breast milk to their infants and used formula or other alternatives exclusively [12]. This classification allowed us to analyze variations in water balance and intake patterns among the different breastfeeding practices Approaching and recruiting potential participants from Athens, Greece was conducted through social media outreach and by providing the study link in local pediatricians’ and gynecologists’ offices. Each potential participant received detailed explanation about the study’s objectives and procedures. Informed consent was obtain from all participants before they engaged in the study. The survey, which was conducted in winter, was self-administered via Google forms. The study received approval from the Bioethics Committee of the Agricultural University of Athens, Greece (approval number: 34/27-7-2020).

### 2.2. Questionnaires

To estimate water balance in lactating women, the WBQ-N was employed [10]. The WBQ-N is an adoption of the Water Balanced Questionnaire (WBQ) [9], specifically modified to include questions that reflect the unique dietary patterns, fluid intake behaviors, and unique hydration needs of breastfeeding women [10,11]. The WBQ-N was validated in lactating women consists of seven parts: 1. demographic and socio-economic data, including anthropometric characteristics (weight, height, age), 2. lifestyle and health characteristics (medications, supplements, disease, type of breastfeeding), 3. physical activity: assessed using the International Physical Activity Questionnaire [14], 4. semi-quantified food frequency diary: recording intake of 58 types of food based on their water content, adapted from United States Department of Agriculture (USDA) data for research purposes [11], 5. liquid and drink intake: recording of daily water intake (in mL of water per day), 6. water loss: assessing water loss through sweat (rated in a scale from “1” for minimal to “10” for maximal during exercise and rest), urine (frequency ranging from 1 f/day to 10 or more/day), feces (frequency ranging from 1/day and up to 1/10 days), and breastfeeding (frequency and duration), and 7 hydration habits and knowledge: evaluating practices related to optimal hydration and understanding of appropriate daily water intake.

### 2.3. Evaluation of Water Intake, Loss and Balance

The estimation of the total water intake from the 58 specific types of food in the WBQ-N was performed using data from the USDA database [15]. Water from food and drinking water were evaluated as separate variables [10]. Total water intake was calculated by summing water intake from drinking water (only), water intake from liquids (except for water), and water intake from foods. Water intake from liquids (except for water) includes all liquids not individually reported in the table, such as energy drinks, alcohol.

Water from sweat loss was calculated separately for intensive, moderate-intensive and sedentary physical activities. Participants assessed sweat loss using a 10-point scale: for intensive exercise, 1 point equaled 1000 mL/h and 10 points equaled 2000 mL/h. For moderate-intensity exercise, 1 point equaled 400 mL/h and 10 points equaled 700 mL/h. For sedentary behaviors, 1 point equaled 0.01 mL/h and 10 points equaled 0.02 mL/h. Urinary water loss was assessed based on a 5-point scale: 1 point (urinating 1 time/day) equaled 600 mL/day, and 5 points (urinating more than 10 times/day) equaled 3000 mL/day. Fecal water loss was estimated using a 5-point scale: 1 point (more than 1 bowel movement/day) equaled 200 mL/day, and 5 points (1 bowel movement/10 days) equaled 50 mL/day.

Water balance estimation from the questionnaires was performed by calculating the difference between the “total water intake” and the “total water loss”. Total water intake included water from food, beverages and drinking water, while total water loss included water lost through sweat, urine, and feces.

To estimate of water balance, according to prevailing guidelines, we subtracted “total water intake” from the population-specific recommendations provided by the European Food Safety Authority (EFSA). These guidelines recommend 2700 mL/day for breastfeeding women and 2000 mL/day for mixed and non-breastfeeding individuals [4]. Binary variables were generated to identify instances of negative water balance, assigning a value of 0 to indicate negative water balance, when the water balance was <0, and a value of 1 otherwise (positive water balance).

### 2.4. Statistical Analysis

Normality of continuous variables was assessed using the Shapiro-Wilk test and histograms. Data are presented as absolute (relative) frequency for categorical variables, 5% trimmed mean (IQR) for skewed continuous variables. Differences in proportions between breastfeeding groups were examined with or chi-square test, while differences in continuous variables were assessed using the Kruskal-Wallis test. To investigate the association between quartiles of ‘water balance’ and various anthropometric, demographic and lifestyle descriptors, ordinary logistic regression models were employed. In the context of ordinal regression, we tested the parallelism hypothesis to verify that the relationships between predictor variables and the ordinal outcome variable (water balance) were consistent across all levels of the outcome variable. Reported *p*-values are based on two-sided tests, with statistical significance at a = 5%.

All statistical analyses were performed using R Core Team (2021). R: A language and environment for statistical computing. R Foundation for Statistical Computing, Vienna, Austria. Available at https://www.R-project.org/, R version 4.1.2 (accessed on 1 November 2021), and RStudio Team (2020). RStudio: Integrated Development for R. RStudio, PBC, Boston, MA, USA. Available at http://www.rstudio.com/ accessed on 1 February 2024.

## 3. Results

Sociodemographic and anthropometric characteristics were examined across different breastfeeding groups: exclusive, mixed, and no breastfeeding (Table 1). A total of 529 mothers participated in the study, with varying distributions across the breastfeeding groups: 210 (39.7%) in the exclusive breastfeeding group, 196 (31.9%) in the mixed breastfeeding group, and 150 (28.4%) in the no breastfeeding group.

Comparison of weight and BMI among the breastfeeding groups showed no statistically significant differences (*p* = 0.548 and *p* = 0.931, respectively). Similarly, the proportions of mothers classified as underweight, normal weight, overweight, and obese did not significantly differ across the groups (*p* = 0.883). However, there was a statistically significant difference observed in the mothers’ age across the breastfeeding groups (*p* = 0.014). No significant difference was found in mothers’ education level among breastfeeding groups (*p* = 0.506).

Total water intake and water intake from drinking water did not significantly differ among breastfeeding groups (*p* = 0.907, *p* = 0.089), but water intake from liquids (excluding drinking water) revealed a significant difference between groups (*p* < 0.001). Water intake from juices also exhibited a significant difference among the groups (*p* = 0.019), with mothers in the exclusive breastfeeding group consuming the highest amount (mean = 103 mL). Additionally, significant differences were observed in water intake from coffee (*p* < 0.001) and milk (*p* = 0.015) among the breastfeeding groups.

Total water loss varied significantly among the groups (*p* < 0.001), with mothers in the exclusive breastfeeding group to have the highest total water loss (5% trimmed mean = 3888.10 mL). The analysis of water loss from urine, feces and sweat revealed no significant differences across breastfeeding groups (*p* = 0.755). Water balance significantly differed among the groups (*p* < 0.001), with mothers in the exclusive breastfeeding group exhibiting the lowest water balance (5% trimmed mean = −475.36 mL), indicating a higher deficit compared to the other groups (Table 2).

The association between various sociodemographic, anthropometric characteristics, and water consumption patterns among women across different breastfeeding groups revealed significant differences in the proportion of mothers with adequate total water intake based on recommendations across exclusive breastfeeding, mixed breastfeeding, and no breastfeeding groups (*p* < 0.001). Although, no significant differences were found in weight, BMI and mothers’ education among the groups, slight variations in mothers’ age were noted, with those in the no breastfeeding group tending to be slightly older (*p* = 0.048) (Table 3). No significant differences were found in total water intake and water intake from drinking water among the breastfeeding groups. However, notable variations were observed in water intake from liquids except for water, particularly in juices (*p* = 0.024) and coffee (*p* < 0.001). Exclusive breastfeeding mothers tended to consume a higher amount of water from juices and a lower amount of coffee compared to mixed and non-breastfeeding mothers. Significant differences were also noted in total water loss and water balance among the groups, with exclusive breastfeeding mothers to exhibit the highest total water loss and the lowest water balance, indicating a potential risk of dehydration (*p* < 0.001). However, results show that water loss from urine, feces, and sweat does not significantly differ across breastfeeding groups (*p* > 0.05).

Table 4 presents the distribution of participants and their water balance, intake, and loss categorized into quartiles among breastfeeding women. Significant differences were observed in the distribution of participants across quartiles of water balance among exclusive breastfeeding, mixed breastfeeding, and no breastfeeding groups (*p* < 0.001). For exclusive breastfeeding, the majority of participants were in the first quartile (*n* = 66, 31.40%), indicating lower water balance, while for mixed breastfeeding the majority of participants were in the Q1–Q3 and for the no breastfeeding the majority of participants were in the last quartile (*n* = 55, 36.7%). Significant differences were observed in the water intake from liquids except for water, particularly in the exclusive breastfeeding group, where higher intake was associated with higher quartiles (*p* < 0.001). Similar trends were observed for water intake from drinking water, with significantly higher intake in higher quartiles for all breastfeeding groups (*p* < 0.001). Water loss showed significant variations across quartiles for all breastfeeding groups (*p* < 0.05). Exclusive breastfeeding mothers exhibited the highest water loss in the first quartile, gradually decreasing in higher quartiles. The analysis of water loss from urine revealed a decrease across the quartiles, yet these differences were not statistically significant (*p* = 0.098). No significant differences were observed in water loss from feces across quartiles (*p* = 0.878). Water loss from sweat exhibited a substantial and statistically significant decrease across the quartiles (*p* < 0.001). Conversely, mixed breastfeeding and no breastfeeding groups showed a decrease in water loss from the first to the fourth quartile.

The results from ordinary logistic regression revealed several significant associations between predictor variables and quartiles of water balance (Table 5). Exclusive breastfeeding compared to no breastfeeding was significantly associated with lower water balance across all quartiles (*p* < 0.001). Moreover, water intake from drinking water, water from foods, coffee and milk showed significant positive associations with water balance, while water from Isotonic/energy drinks showed significant negative associations with water balance.

## 4. Discussion

Hydration during breastfeeding is essential for both the mother and the baby, as adequate water intake is crucial for maintaining sufficient milk production. This study examined water consumption patterns and estimated water balance among breastfeeding women from a European population engaged in different feeding practices. Our analysis revealed minimal differences in water loss from urine, feces, and sweat across breastfeeding groups, suggesting that breastfeeding status does not significantly influence these specific water loss pathways. Significant differences were found in water consumption patterns among breastfeeding groups. Exclusive breastfeeding mothers predominantly fell into the lower quartiles of water balance, indicating a lower water balance compared to mixed and non-breastfeeding mothers.

In our study we found that water loss patterns varied significantly across quartiles of water balance and breastfeeding methods. Total water loss differed markedly across quartiles and breastfeeding groups, with exclusive breastfeeding mothers showing distinct water loss characteristics. Despite uniform water loss from urine and feces across exclusive, mixed, and non-breastfeeding groups, water loss from sweat exhibited notable variability across quartiles. Specifically, we found that exclusive breastfeeding mothers had higher levels of sweat loss, potentially reflecting increased physiological demands and metabolic changes related to lactation. This suggests that factors other than breastfeeding status might be more influential in determining these losses. This variation could be influenced by individual differences, such as individual metabolism or dietary intake, physical activity or environmental conditions affecting sweat production [5,8]. The highest levels of sweat loss in the exclusive breastfeeding group may reflect their increased physiological demands and metabolic changes related to lactation, which could elevate sweat production as a means to regulate body temperature [1,12,13].

The findings of the current study revealed several anthropometric factors to be associated with water balance. We found that BMI of mothers was positively associated with higher quartiles of water balance, indicating that larger body mass may be linked to greater water needs and intake, possibly driven by higher metabolic demands. Age also played a pivotal role, with older mothers more likely to be in higher quartiles of water balance, which might reflect more established health routines or greater experience in managing hydration needs effectively [1,4,6,7].

Our findings also revealed variations among breastfeeding groups regarding their water consumption patterns. While total water intake and intake from drinking water did not differ significantly among groups, exclusive breastfeeding mothers seemed to consume significantly lower amounts of water from beverages except for water compared to mixed and non-breastfeeding mothers. This discrepancy could be attributed to several factors. Exclusive breastfeeding mothers may prioritize plain water over other beverages due to heightened awareness of hydration needs directly tied to lactation, potentially influenced by guidance from healthcare providers. Moreover, they might avoid certain beverages due to concerns about their impact on milk production or their composition, which could affect the breastfeeding infant. For instance, caffeine and high-sugar beverages are often avoided due to concerns about their effects on the baby and milk supply [1,12]. In addition, while total water intake did not differ significantly among quartiles of water balance within exclusive and mixed breastfeeding groups, differences were again observed in water intake from beverages and drinking water. Exclusive breastfeeding mothers consumed more water from beverages and drinking water as quartiles of water balance increased. This suggests that water intake from beverages could help exclusive breastfeeding mothers to meet the elevated hydration requirements achieving higher quartile of water balance. However, the current literature indicates that replacing water with sugar-sweetened beverages, juice, and milk is associated with higher energy intake [8,16,17]. Therefore, while diverse fluid consumption can aid hydration, it is essential for mothers to balance this with maintaining a healthy energy intake and nutritional quality to support both their well-being and effective lactation.

As far as the water from sources apart from liquids is considered, the proportion obtained from food varies mainly based on the amount of fruit and vegetable consumed. It is estimated that about 22% of water in the United States comes from food intake [18], whereas it would be much higher in European countries, because of higher consumption of fruit and vegetable [19,20]. More specifically, one comprehensive study of water intake in pregnant and lactating women in the United States discovered that a 20.7% of daily water intake came from food water content; however, this research was dependent on a poor overall assessment of water intake [21]. In our study, although there was no difference in the water from foods between the breastfeeding groups, it accounted for approximately 23% of total water intake in all groups and was a significant predictor of water balance, which is inline while the above-mentioned literature.

We found significant variations in water loss with exclusive breastfeeding mothers to have the highest amount. In addition, breastfeeding method had a marked impact on estimated water balance, with exclusive breastfeeding mothers to exhibit the lowest balance and potentially higher dehydration risk. This aligns with previous research in other non-European countries [5,6,7,8,22,23], suggesting that this population is in high risk of dehydration and probably the feeling of thirst is not enough for achieving adequate hydration; even in the climate conditions are not such extreme.

In our study, a substantial portion of the mixed breastfeeding group was in a state of negative water balance. Mixed breastfeeding is frequently overlooked in nutritional research and lacks clear and specific water intake recommendations [24,25]. This finding underlines the need for further research and targeted guidelines tailored to the unique requirements of individuals engaged in mixed breastfeeding. The absence of precise recommendations for this specific demographic poses potential health risks, as evidenced by the prevalence of negative water balance within our study [26,27]. Given the variations in water consumption patterns and hydration risks identified in our study, practical strategies to improve water intake among breastfeeding women are essential. 

Although our study conducting during wither, seasonal variations play a critical role in determining the fluid needs of breastfeeding women. For example, during summer, increased temperatures and humidity can lead to higher water loss through perspiration, necessitating greater fluid intake to prevent dehydration. In contrast, in winter, the reduced sensation of thirst may result in lower water consumption, despite indoor heating systems creating a dry environment that contributes to water loss through the respiratory system and skin [18,22]. Healthcare providers should offer tailored hydration advice for breastfeeding women, adjusting for seasonal variations to ensure adequate hydration year-round. Strategies such as consuming hydrating foods in summer and setting reminders for regular water intake in winter can help mitigate these seasonal risks.

We recognized that there were some constraints in this study. Firstly, this was a cross-sectional survey, and as such findings could not be generalised in other populations [28]. Also, the tools (diaries and questionnaires) used could increase risk for recalling bias about dietary and water intake information [29]. Furthermore, within the scope of this investigation, the determination of water balance for the mixed breastfeeding population was derived from recommendations intended for the general public. This approach may have introduced an element of overestimation into our calculations. Another limitation, is that we simplified the physical activity items based on the standard questionnaire (IPAQ) rather than using actual meters e.g., pedometers [30,31].

Despite these limitations, the current survey had some advantages: to our best knowledge, this is the first survey on assessing water intake in this specific population in Greece and includes data on actual water intake as well as on water from foods and beverages. Also, we compared water intake both based on current recommendation and with actual estimations for water balance. Furthermore, the sample size was adequate with a satisfactory number of women performing different feeding styles, which allowed creating groups with satisfactory number of participants for statistical tests. Finally, our models have been controlled for many factors associated with diet behaviors in order to reduce any confounding effect. Considering the paramount importance of optimal hydration during lactation, our survey findings could serve as foundational empirical data, urging a reconsideration and refinement of the existing recommendations concerning water intake for these distinct populations. This underscores the necessity for an updated and more accurate assessment of the water needs of postpartum women, especially those engaged in breastfeeding, to better guide health and nutritional guidelines.

## 5. Conclusions

In summary, our findings reveal significant variations in water consumption patterns across different breastfeeding practices, highlighting the need for tailored hydration guidelines for breastfeeding women. Exclusive breastfeeding mothers tend to consume more plain water and fewer other beverages. This behavior underscores the importance of educating breastfeeding mothers about optimal fluid intake, considering both water and other beverages to support lactation effectively.

Understanding the patterns of water intake and its relationship with breastfeeding practices is crucial for developing specific recommendations that cater to the unique hydration needs of breastfeeding mothers. Future research should explore the long-term health implications of hydration status on both maternal and infant outcomes, considering the potential risks of high energy intake from certain beverages. It is also essential to develop more accurate and individualized methods for assessing water balance in breastfeeding women, taking into account the role of environmental factors, such as climate and seasonal variations in fluid needs.

## Figures and Tables

**Table 1:** Sociodemographic and anthropometric characteristics in mothers per breastfeeding group.

	Total	Exclusive	Mixed	No Breastfeeding	*p*-Value
Mothers *n* (%)	529	210 (39.7)	196 (31.9)	150 (28.4)	
Weight (kg)	67.6 (20.0)	67.0 (19.0)	67.3 (18.8)	68.7 (20.0)	0.548
BMI of mothers (kg/m^2^)	24.5 (6.6)	24.4 (6.0)	24.6 (6.6)	24.1 (6.5)	0.931
Underweight *n* (%)	9 (1.7)	5 (0.9)	3 (0.6)	1 (0.2)	0.883 *
Normal weight *n* (%)	310 (58.6)	126(23.8)	99 (18.7)	85 (16.1)	
Overweight *n* (%)	121 (22.9)	45(8.5)	38 (7.2)	38 (7.2)	
Obese *n* (%)	89 (16.8)	34 (6.4)	29 (5.5)	26 (4.9)	
Mothers’ age (years)	34.1 (6.0)	34.2 (6.0)	33.3 (6.0)	35.0 (6.0)	0.014
Mothers’ education (years of school)	16.6 (2.0)	16.7 (2.0)	16.5 (2.0)	16.5 (2.0)	0.506

Results are presented as the 5% trimmed mean (IQR) or *n* (%). *p*-values refer to comparisons between groups and are derived via Kruskal-Wallis test or chi-square test *.

**Table 2:** Characteristics of water consumption in mothers per breastfeeding group.

(mL/day)	Total	Exclusive	Mixed	No Breastfeeding	*p*-Value
Total water intake	3125.4 (1178.3)	3214.8 (1145.7)	3115.4 (1151.8)	3079.0 (1292.1)	0.907
Water intake from drinking water (only)	1760.89 (724.36)	1845.99 (738.72)	1714.88 (685.47)	1691.92 (739.06)	0.089
% of total water intake	50.24	57.2	55.1	55.2	
Water intake from liquids (except for water)	799.7 (658.1)	818.3 (759.2)	862.0 (433.2)	684.0 (86.7)	<0.001
% of total water intake	25.6	19.7	21.7	22.1	
Water from foods	723 (279)	744 (307)	724 (234)	700 (279)	0.157
% of total water intake	24.16	23.1	23.2	22.7	
Water from juices	79 (244)	103 (244)	87 (244)	54 (35)	0.019
% of total water intake	2.5	3.2	2.8	1.7	
Water from soft drinks	48 (38)	56 (38)	49 (38)	38 (38)	0.647
% of total water intake	1.5	1.7	1.6	1.2	
Water from tea	63 (29)	60 (29)	60 (29)	77 (29)	0.902
% of total water intake	2.0	1.9	1.9	2.5	
Water from coffee	356 (475)	330 (475)	316 (475)	444 (297)	<0.001
% of total water intake	11.4	10.3	10.2	14.4	
Water from milk	104.0 (240.6)	84 (241)	162 (241)	71 (34)	0.015
% of total water intake	3.3	2.6	5.2	2.3	
Total water loss	3515.24 (1356.68)	3888.10 (1297.81)	3612.64 (1334.41)	2887.51 (1244.12)	<0.001
Water loss from urine	1486.02 (450)	1482.50 (900)	1475 (850)	1506.47 (900)	0.755
Water loss from faces	170.70 (50)	168.06 (50)	175.57 (50)	169.09 (52)	0.635
Water loss from sweat	941.61 (1648.67)	916.53 (1563.38)	926.19 (1710.88)	996.14 (1763.33)	0.491
Water balance	−87.52 (1450.61)	−475.36 (1526.08)	−209.31 (1352.92)	580.15 (1199.98)	<0.001

Results are presented as the 5% trimmed mean (IQR). *p*-values refer to comparisons between groups and are derived via Kruskal-Wallis test.

**Table 3:** Association of sociodemographic and anthropometric characteristics in women with adequate total water intake based on recommendations.

	Exclusive	Mixed	No Breastfeeding	*p*-Value
Mothers *n* (%) who met the recommendation	160 (76.2)	122 (62.2)	131 (87.3)	0.004
Weight (kg)	67.2 (18.0)	67.9 (19.0)	69.0 (20.0)	0.533
BMI of mothers (kg/m^2^)	24.4 (6.1)	24.1 (7.2)	24.7 (6.3)	0.797
Underweight *n* (%)	2 (0.5)	1 (0.2)	1 (0.2)	0.983 *
Normal weight *n* (%)	96 (23.2)	71 (17.2)	73 (17.7)	
Overweight *n* (%)	36 (8.7)	27 (6.5)	33 (8.0)	
Obese *n* (%)	26 (6.3)	23 (5.6)	24 (5.8)	
Mothers’ age (years)	34.2 (6.0)	33.6 (5.8)	35.2 (6.0)	0.048
Mothers’ education (years of school)	16.7 (2.0)	16.6 (2.0)	16.4 (2.0)	0.407
Total water intake (mL/day)	3635.9 (1000)	3648.9 (750)	3494.1 (1050)	0.117
Water intake from drinking water (only) (mL/day)	1986.1 (945)	1746.2 (760)	1810.8 (960)	0.095
Water intake from liquids (except for water) (mL/day)	862.1 (505.4)	823.50 (716.3)	892.62 (438.12)	<0.001
Water from foods (mL/day)	762.5 (339.0)	741.6 (242.0)	715.2 (278.0)	0.215
Water from juices (mL/day)	110.1 (244.2)	91.5 (244.2)	53.3 (34.9)	0.024
Water from soft drinks (mL/day)	47.1 (37.9)	50.6 (37.9)	41.7 (37.9)	0.896
Water from tea (mL/day)	69.4 (74.8)	67.5 (29.9)	82.5 (29.9)	0.524
Water from coffee (mL/day)	334.9 (43.0)	352.6 (475.0)	461.9 (475.0)	<0.001
Water from milk (mL/day)	94.8 (240.6)	160.6 (240.6)	74.5 (34.4)	0.087
Total water loss (mL/day)	3790.47 (1680)	3508.38 (1470.1)	2767.07 (1749.2)	<0.001
Water loss from urine	1600 (563)	1475 (1350)	1808.33 (675)	0.755
Water loss from faces	172.22 (63)	188.89 (56)	127.04 (78)	0.635
Water loss from sweat	1232.86 (1534.17)	1519.48 (1509.5)	1307.01 (1223.25)	0.491
Water balance (mL/day)	−128.60 (1869.3)	−120.05 (1643.5)	715.86 (1330)	<0.001

Adequate total water intake based on daily recommendation: 2700 mL for breastfeeding and 2000 mL for non-breastfeeding women. Results are presented as the 5% trimmed mean (IQR) or *n* (%). *p*-values refer to comparisons between groups and are derived via Kruskal-Wallis test or chi-square test *.

**Table 4:** Distribution of participants, water balance, intake and loss in breastfeeding groups.

	Quartiles of Water Balance (mL/day)				
(mL/day)	1st Quartile (Q1) (<−994.47 mL)	2nd Quartile (Q2) (−994.47 to 33.31 mL)	3rd Quartile (Q3) (33.31 to 921.24 mL)	4th Quartile (Q4) (>921.24 mL)	*p*-Value
Exclusive, *n* = 210 (39.7%)	66 (31.40%)	50 (23.80%)	37 (17.60%)	37 (17.60%)	<0.001
Total water intake	1996.77 (983.38)	1760.00 (920.06)	1899.71 (1117.16)	2082.50 (784.02)	0.437
Water intake from liquids (except for water)	617.10 (385.25)	772.51 (399.88)	900.57 (401.87)	982.73 (426.56)	<0.001
Water from drinking water (only)	1611.52 (741.22)	1716.73 (699.24)	1887.78 (16.02)	2256.76 (690.08))	<0.001
Water from foods	734.24 (243.64)	796.72 (307.55)	719.52 (217.05)	876.29 (376.27)	0.058 *
Water loss	5103.92 (1007.05)	3760.85 (727.55)	3150.64 (724.56)	2581.80 (793.42)	<0.001
Mixed, *n* = 169 (31.9%)	37 (25.30%)	43 (25.40%)	39 (23.10%)	27 (16.00%)	<0.001
Total water intake	1704.32 (743.04)	1575.81 (781.45)	1666.58 (987.90)	1796.92 (676.88)	0.737
Water intake from liquids (except for water)	783.19 (440.53)	735.23 (372.43)	763.81 (458.40)	1048.53 (274.82)	0.014
Water from drinking water	1482.70 (682.41)	1593.02 (565.40)	1689.74 (691.94)	2080.74 (457.57)	0.003
Water from foods	652.90 (235.45)	757.58 (290.26)	824.21 (273.90)	853.27 (385.34)	0.028
Water loss	4948.14 (1237.74)	3522.31 (817.32)	2900.63 (742.60)	2621.46 (581.06)	<0.001
No breastfeeding, *n* = 153 (28.4%)	15 (10.0%)	26 (17.3%)	41 (17.3%)	55 (36.7%)	<0.001
Total water intake	1436.00 (946.50)	1618.75 (787.63)	1736.75 (1088.06)	2033.85 (1036.96)	0.131
Water intake from liquids (except for water)	858.94 (386.91)	708.26 (350.05	836.43 (333.34)	936.43 (283.49)	0.012
Water from drinking water (only)	1617.33 (776.36)	1440.77 (839.16)	1600.00 9617.77)	1730.00 (702.50)	0.002
Water from foods	641.53 (225.33)	730.26 (231.39)	704.36 (253.36)	775.17 (249.68)	0.240
Total water loss	4829.96 (1149.91)	3251.80 (992.94)	2740.17 (915.21)	2135.88 (579.07)	<0.001
Water loss from urine	1555.51 (381.16)	1487.61 (450)	1448.72 (450)	1229.58 (454)	0.098
Water loss from faces	169.26 (50)	172.41 (54)	172.79 (50)	166.32 (52)	0.878
Water loss from sweat	2177.35 (1976.71)	887.26 (1507.67)	565.70 (979.83)	246.34 (479)	<0.001

Results are presented as P50 (P25, P75), as indicated for skewed variables. Quartiles of water balance were defined according to water balance of non-pregnant women. *p*-values are derived through the Kruskal–Wallis H-test for the skewed variables. * marginally significant

**Table 5:** Results from ordinary logistic regression for examination of factors in association with quartiles of water balance.

	Estimate (95% CI) of Predictor Variable	*p*-Value	Estimate (95% CI) of Quartile		*p*-Value
BMI of mothers (kg/m^2^)	0.027 (−0.01, 0.60)	0.111	Q1	−0.42 (−1.28, 0.44)	0.341
			Q2	0.69 (−0.17, 156)	0.116
			Q3	1.78 (0.91, 2.66)	<0.001
Mothers’ age (years)	0.02 (−0.02, 0.05)	0.371	Q1	−0.59 (−1.74, 0.57)	0.320
			Q2	0.52 (−0.63, 168)	0.374
			Q3	1.61 (0.45, 2.77)	0.007
Mothers’ education (years of school)	−0.03 (−0.10, 0.03)	0.276 (I)	Q1	−0.59 (−1.74, 0.57)	0.320
			Q2	0.52 (−0.63, 168)	0.374
			Q3	1.61 (0.45, 2.77)	0.007
Exclusive breastfeeding vs. no	−1.263 (−1.67,−0.85)	<0.001	Q1	−1.98 (−2.34, −1.62)	<0.001
Mixed breastfeeding vs. no	−0.99 (−1.42, −0.57)	<0.001	Q2	−0.81 (−1.13, −0.48)	<0.001
			Q3	0.35 (0.04, 0.67)	0.030
Water from drinking water (mL/day)	0.01 (0.00, 0.02)	0.002	Q1	−0.37 (−0.86, 0.11)	0.129
			Q2	0.74 (0.26, 1,22)	0.003
			Q3	1.84 (1.33, 2.35)	<0.001
Water from foods (mL/day)	0.01 (0.00, 0.02)	0.002	Q1	−0.37 (−0.86, 0.11)	0.129
			Q2	0.74 (0.26, 1,22)	0.003
			Q3	1.84 (1.33, 2.35)	<0.001
Water from juices (mL/day)	0.01 (−0.01, 0.01)	0.242	Q1	−1.04 (−1.27, −0.81)	<0.001
			Q2	0.07 (−0.14, 0.28)	0.541
			Q3	1.15 (0.92, 1.39)	<0.001
Water from soft drinks (mL/day)	0.01 (−0.01, 0.01)	0.720	Q1	−1.09 (−1.31, −0.86)	<0.001
			Q2	0.02 (−0.18, 0.22)	0.851
			Q3	1.11 (0.88, 1.33)	<0.001
Water from tea (mL/day)	0.01 (0.01, 0.01)	<0.001 (I)	Q1	−0.91 (−1.13, −0.68)	<0.001
			Q2	0.24 (0.03, 0.45)	0.023
			Q3	1.37 (1.13, 1.62)	<0.001
Water from coffee (mL/day)	0.01 (0.01, 0.01)	0.056 *	Q1	−0.88 (−1.19, −0.57)	<0.001
			Q2	0.23 (−0.07, 0.53)	0.132
			Q3	1.32 (1.00, 1.65)	<0.001
Water from milk (mL/day)	0.01 (0.01, 0.01)	0.004	Q1	−0.96 (−1.19, −0.73)	<0.001
			Q2	0.16 (−0.05, 0.37)	0.134
			Q3	1.26 (1.02, 1.50)	<0.001
Water from Milkshakes/sherbets (mL/day)	−0.02 (−0.06, 0.02)	0.233	Q1	−1.47 (−2.11, −0.83)	<0.001
			Q2	−0.36 (−0.99, 0.26)	0.256
			Q3	0.73 (0.09, 1.36)	0.024
Water from Isotonic/energy drinks (mL/day)	−0.01 (−0.10, −0.01)	0.037	Q1	−1.21 (−1.44, −0.98)	<0.001
			Q2	−0.09 (−0.29, 0.11)	0.385
			Q3	1.00 (0.78, 1.22)	<0.001
Water from Alcoholic drinks (mL/day)	−0.01 (−0.01, 0.01)	0.415	Q1	−1.14 (−1.37, −0.91)	<0.001
			Q2	−0.04 (−0.24, 0.17)	0.736
			Q3	1.05 (0.82, 1.28)	<0.001

(I) Include interaction terms between the predictor variable and the categories of the outcome variable. * marginally significant

## Data Availability

The data presented in this study are available on request from the corresponding author. The data are not publicly available due to the confidential nature of some information.
